# Impact of the Refugee Crisis on the Greek Healthcare System: A Long Road to Ithaca

**DOI:** 10.3390/ijerph15081790

**Published:** 2018-08-20

**Authors:** Ourania S. Kotsiou, Panagiotis Kotsios, David S. Srivastava, Vaios Kotsios, Konstantinos I. Gourgoulianis, Aristomenis K. Exadaktylos

**Affiliations:** 1 Respiratory Medicine Department, Faculty of Medicine, University of Thessaly, Biopolis, 41500 Larissa, Greece; kgourg@med.uth.gr; 2 International Business Department, Perrotis College, 57001 Thessaloniki, Greece; panagiotiskotsios@gmail.com; 3 Inselspital, University Hospital Bern, 3010 Bern, Switzerland; DavidShiva.Srivastava@insel.ch (D.S.S.); Aristomenis.Exadaktylos@insel.ch (A.K.E.); 4 Metsovion Interdisciplinary Research Center, National Technical University of Athens, 44200 Athens, Greece; vaioskotsios@gmail.com

**Keywords:** economic crisis, Greece, migration, National Health System, refugee

## Abstract

Greece is the country of “Xenios Zeus”, the Ancient Greek god of foreigners and hospitality; however, it is also the main point of entry to Europe. Since the beginning of 2014, 1,112,332 refugees crossed the borders of Greece. Overall, 33,677 children and adolescent refugees sought asylum in Greece from 2013 to 2017, while 57,042 refugees are currently being hosted. The rapid entry of refugees into Greece raised the critical issue of health policy. The Greek National Health Service (NHS) faces many challenges. Adequate economic and human support is essential if this situation is to be managed successfully. However, Greece still bears the burden of the economic downturn since 2009. In fact, the crisis led to shortages in crucial equipment, and unmet health needs for both locals and refugees. The NHS deals with traumatic experiences, as well as cultural and linguistic differences. Overcrowded reception centers and hotspots are highly demanding and are associated with severe disease burden. This highlights the importance of guidelines for medical screening, healthcare provision, and a well-managed transition to definitive medical facilities. Furthermore, non-governmental organizations make an essential contribution by ensuring appropriate support to refugee minors, especially when they experience poor access to the NHS.

## 1. Introduction

The refugee crisis resulted in journeys of despair to and throughout Europe and around the world. By the end of 2016, the number of forcibly displaced people reached a record of 65.6 million worldwide [1]. The estimates indeed reached the highest level since the end of World War II [1]. This population comprises refugees (22.5 million), persons internally displaced within their own countries (40 million), and asylum seekers (3.1 million) [1]. Based on the current world population of 7.4 billion, this would mean that one in every 113 people is either a refugee, an internally displaced person, or an asylum seeker [2]. More importantly, 10 million people around the world are stateless and denied access to mobility and fundamental rights, such as education, healthcare, and employment [1,2].

Over the two-year period of 2015 and 2016, 2.68 million refugees arrived in Europe [3,4]. The vast majority of them entered the European Union (EU) through a combination of land and sea routes [5]. Furthermore, 1,014,973 and 231,075 people in 2015 and in the first six months of 2016, respectively, who were seeking a better and safer life in Europe, risked their lives by attempting to cross the Mediterranean [3]. The majority of these entered Greece (1,015,100), and the rest reached Italy (224,064) and Spain (6884) by sea alone [3]. The year of 2016 was the deadliest for sea crossings, with 5096 deaths reported [4]. In the first half of 2017, over 105,000 people reached Europe [5], while over 2700 people were documented as having lost their lives while crossing the Mediterranean, with unconfirmed reports of many others perishing en route [5]. The ongoing refugee crisis challenges many countries to address the large number of significant political, economic, social, and health dimensions of this humanitarian crisis [6,7,8].

**Figure 1:**
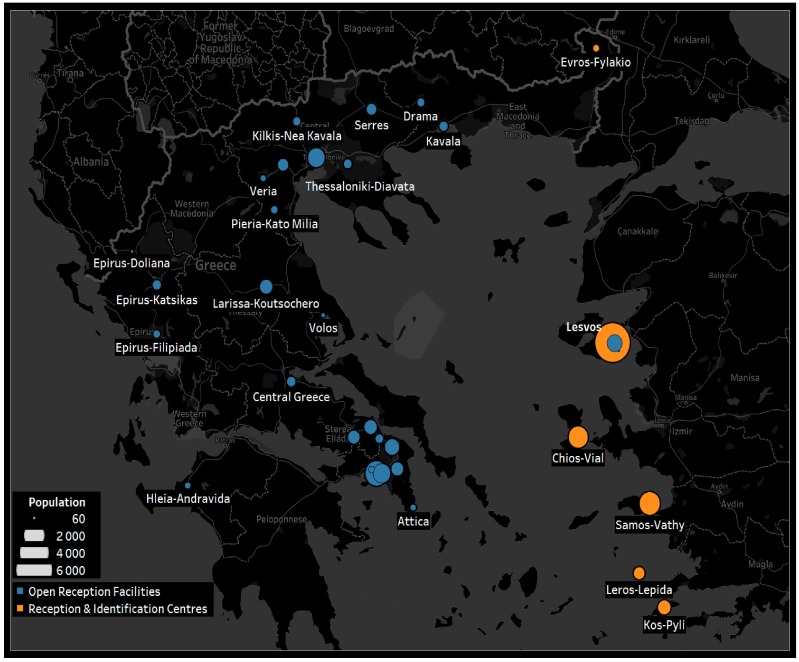
The main reception centers in Greece. Chios, Kos, Lesvos, Leros, Samos, and other Greek islands are rapidly and easily accessible points of entry into Europe. Notes: The orange circles represent the main reception and identification centers in Greek islands; the blue circles represent the open reception facilities (hotspots) on the mainland. The area of the circle depicts the size of the refugee population.

From 2015 to 2016, Greece experienced an unprecedented influx of refugees and migrants fleeing their home countries in the Middle East because of war [3,6,7,8,9]. Since the beginning of 2014, a total of 1,112,332 refugees arrived by sea in Greece [6]. Nearly 857,000 people arrived in Greece in 2015, a 750% increase from 2014 (41,038 arrivals) [3,6]. Approximately 16,000 sea arrivals were recorded in the first six months of 2018 [6]. The refugees mainly landed on Greek islands, which constitute a border with quick and easy access into Europe (Figure 1) [6,9]. From 2014 to 2017, the United Nations High Commissioner for Refugees (UNHCR) unfortunately recorded at least 1700 people dead or missing on the treacherous crossing from Turkey to Greece along the Eastern Mediterranean route [6,7,8]. Greece currently hosts approximately 57,000 refugees, 60% of whom are estimated to live on the mainland and the rest in the main reception centers in the islands of Lesvos, Chios, Kos, Samos, and Leros, or scattered throughout Greece [6]. More than 3000 are traveling alone [3].

However, Greece is in the middle of a severe economic crisis and is struggling to pay for the migrant movement. A substantial part of the country was already profoundly affected by the economic downturn [10]. There are virtually no opportunities for employment that promise financial stability, which could then facilitate integration into the new country [11,12,13]. Pajic et al. recently highlighted the potentially adverse effect of social barriers in searching for a job [13]. Poor living conditions in Greece led the majority of refugees to travel deeper into Europe [14]. The main destination countries are Germany, Austria, and Sweden [2,4]. However, the barriers recently set up by many countries, notably the Former Yugoslav Republic of Macedonia (FYROM; soon to be Northern Macedonia), make migration much more difficult than in previous years [2,4,14]. In fact, it was documented that almost 60,000 asylum seekers are currently stuck in Greece due to closed European borders [2,14]. It is estimated that refugees now constitute nearly 10% of the Greek population [2,14].

Indeed, the pressure of migration waves became so great that the situation in Greece was described by the UNHCR as resembling a humanitarian crisis [9]. The unprecedented rise in the number of asylum seekers and migrants entering the country had major economic and social consequences for health outcomes [10,15,16,17], and created significant challenges for the Greek National Health Service (NHS) [10,15,16,17]. Public health risks arise both from health conditions during the journey and from health problems in the host country after arrival, along with the need to address the ongoing economic crisis and the fact that there is little pre-existing experience in Greece in the reception and integration of refugees [10,15,16,17]. The aim of the present manuscript is to provide a narrative review of the health conditions of arriving refugees, and the impact of both refugees and their diverse health conditions on the Greek healthcare system. A computer-based search of the English literature was performed in PubMed and Scopus. We limited our research in these databases to the English literature, since resources in other languages were limited. Google Scholar was also used to verify the results of the database searches. A considerable body of information examined in this article includes data that were largely, but not exclusively, presented in Greek studies and Greek gray literature. Key search terms included asylum seekers, children, crisis, Greece, healthcare system, health, immigrants, mental health, migration, National Health System, refugee, refugee crisis, and violence. No filters were applied for the article type and publication dates. Lastly, the reference lists of the retrieved articles were also reviewed for relevant articles, in order to assess whether database results were exhaustive. The literature search was conducted between 1 May and 4 August 2018.

## 2. Health Problems Facing Refugees

### 2.1. Health Problems Facing Adult Refugees

#### 2.1.1. Mental Health and Deficiencies in Psychosocial Support

The refugees who reach resettlement countries were found to have experienced adverse events associated with forced migration, living in displacement under poor circumstances, legal insecurity, detention and deportation, financial privation, social isolation, racism, communication barriers, and employment difficulties, even after they obtained legal permission to remain in the host country [18,19]. These factors place them in jeopardy of mental illness, such as post-traumatic stress disorder (PTSD), depression, and anxiety disorders, which may be persistent [7,18,19,20,21,22,23,24]. It was recently reported that the vast majority of Syrian refugees (up to 92% of 728 individuals) screened positive for anxiety disorder that merited referral for a mental health evaluation [19]. According to Poole et al., a major depressive disorder is significantly over-expressed in female Syrian refugees in Greece, and is associated with large families and the extended asylum procedure [25]. Furthermore, refugees are at an elevated risk of psychopathology, psychosis, schizophrenia, and suicidal tendencies [7,18,19,20,21,22,23,24]. 

Moreover, there is drug abuse and alcoholism among refugees that can subsequently trigger aggressive behavior and exposure to violence [24,26]. Ben Farhat et al. reported that between 31% and 78% of refugees reported having experienced at least one incident of sexual or physical violence in Syria, 25–58% during the journey to Greece, and 5–8% in Greek holding centers [19]. More than three-quarters of the respondents aged over 15 years were diagnosed with an anxiety disorder and required referral for mental health evaluation [19]. However, only two-thirds of participants accepted a referral [19].

The importance of mental health services is underestimated throughout the EU [23,24,25,27,28,29]. As in Greece, the mental health of refugees receives little attention [15]. The reasons for this include poor finances and the failure to establish priorities. Screening for psychopathology is undoubtedly a neglected issue [15]. Most refugees claim that they had little or no access to information and assistance in relation to asylum procedures and mental health support [19]. Thus, a vicious cycle is established, since the uncertainty of their social economic and medical status exacerbates their anxiety [19]. As a consequence, many refugees do not want to stay in Greece and wish to continue migrating to Western Europe [19].

#### 2.1.2. Physical Health and Immunization Status

Moreover, almost 40% of refugees contract diseases and illness in transit [16,17]. Many migrants face onerous conditions during migration, such as a lack of sufficient supplies and adequate shelter and hygiene, and this increases the risk of acquiring infectious diseases [16,17,30]. They present with dehydration and physical injuries, nutrition disorders, diarrhea, and tuberculosis, as well as scabies, one of the most prevalent communicable diseases [16,17,30]. There were many reports that refugees from eastern countries exhibit high rates of infection with hepatitis B virus (HBV) [31,32,33,34]. Handwritten notes by Off Track Health—a grassroots charity running Moria Medical Center in Lesvos—revealed that refugees’ most common symptoms upon arrival in a reception center are fever, chills, sore throat, diarrhea, and chest or abdominal pain. Furthermore, a large number of refugees suffer from infections, asthma attacks, bronchiolitis, or trauma-related injuries [34]. 

Health screening in refugees arriving at the Greek border with Turkey showed that respiratory tract infections were the most common medical problem, diagnosed with a prevalence of 23% in 6899 migrants [35,36]. Another study detected respiratory tract infections in 41% of 33,331 patients who accessed care points of entry into Greece and Serbia [16,17,36,37]. Moreover, pregnancy-related issues are often mentioned [25,28,38]. In some cases, young mothers are too stressed to breastfeed their newborns. The nursing volunteers, such as volunteers of the “Save the Children” non-governmental organization (NGO), help these mothers [34]. As far as chronic disease is concerned, the most frequent condition is hypertension, followed by arthritis, diabetes, chronic respiratory diseases, and cardiovascular disease [16,17].

It is also known that immunization coverage is lower in migrants than in native populations [39,40,41]. It was demonstrated that diseases that can be prevented by vaccination cause flare-ups in reception and holding centers [39,42,43,44,45,46,47,48]. Outbreaks are generally more severe in refugee camps [48]. Sharing dormitories, a lack of accessible toilet facilities, poor hygiene conditions, undernourishment, and limited access to medical care were reported as factors contributing to the increased susceptibility to disease [35,49]. Children and the elderly are undoubtedly particularly vulnerable [50,51,52]. In addition, overcrowding in holding/detention centers or refugee camps may contribute to the rapid spread of communicable diseases, such as influenza, varicella, tuberculosis, measles, and meningococcal disease [16,17,39,42,43,44,45,46,47,48,49]. It is predicted that the complication rates for influenza in this setting will be double those in the general population [53]. However, this risk in refugee populations does not imply a risk of ongoing transmission to the hosting community [30,50].

The 2015 World Health Organization (WHO)/UNHCR/United Nations Children’s Fund (UNICEF) joint statement recommended that refugees should be immediately immunized in accordance with the immunization schedule of the country in which they intend to stay for more than a week. Measles and polio vaccinations are generally awarded the highest priority [48,49,53,54]. In the same year, the European Centre for Disease Prevention and Control (ECDC) emphasized that assessment of vaccination status should be considered as a fundamental part of the general health assessment offered to asylum seekers upon arrival [48,50]. Until recently, there were no screening programs for asylum seekers arriving in Greece [32]. Medical screening was only offered to asylum seekers who applied for a work permit [32]. There was no decision by the Ministry of Health to apply mass screening [55]. By the late spring and summer of 2016, a mass vaccination campaign was finally organized by Médecins Sans Frontières (MSF) in collaboration with the Ministry of Health [55]. Greece now offers specific vaccinations according to the national guidelines for immunization, including those against diphtheria/tetanus/pertussis, poliomyelitis, and measles/mumps/rubella [47]. Vaccination services are provided in holding centers and/or community health services. Greece does not deliver vaccinations at the reception sites [47].

### 2.2. Health Problems Facing Refugee Children

#### 2.2.1. Mental Health and Psychosocial Consequences of Child and Adolescent Refugees

From 2013 to 2017, 24,541 children and 9136 adolescent refugees sought asylum in Greece, the majority of whom were boys (50% and 66%, respectively) aged between 14 and 17 [56,57]. It is well established that more children from Middle Eastern countries, such as Syria, Iraq, and Iran, arrived in 2014–2015 than in 2011–2013. On the other hand, the absolute numbers of refugee children from Afghanistan and Pakistan decreased significantly within this period [56,57,58,59,60,61]. 

On the other hand, the actual number of child refugees who reach Greece may be wrongly estimated, as it is very difficult to verify the actual age of these minors, who are frequently not accompanied by their parents [59]. Young refugees in Greece may give their ages wrongly, depending on potential legal benefits [59].

In September 2017, there were almost 2850 unaccompanied children in Greece. Among these, 1096 children were accommodated in 50 shelters, and 240 in eight campuses nationwide [24,60]. Nowadays, more than half the refugee children are on a waiting list for accommodation, as there is a national lack of shelter capacity [62,63]. Many stay in closed reception facilities or police cells, or may be housed alongside adults in various sites, or in street encampments [62,63].

Children face family separation, detention, limited access to education and recreational activities, trafficking, and security problems [64]. Additionally, although exploitation of children is illegal in Greece, it is recognized that they are exposed to a wide range of risks, such as sexual violence, and physical and psychological harm [65]. In the capital of Greece, Athens, sexual exploitation is increasingly observed in many public places, such as parks, squares, and bars. In these places, particularly teenage boys are sexually abused by older men in exchange for money [63,66,67,68,69,70]. Children’s exposure to violence is a critical public health issue, and is reported to be gender-based. Gender-based violence programming is justifiably focused on women and girls in light of their greater vulnerability; however, specific protection of boys must not be overlooked. In this context, females reported greater exposure to neglect, and males reported greater exposure to lifetime sexual violence [63].

Overall, these forms of harm are associated with a range of adverse health consequences among male and female survivors, including PTSD, depression, drug use, cutting behaviors, and suicidal ideation, along with human immunodeficiency virus (HIV) and other sexually transmitted infections [63]. Young refugees are documented with emotional, and cognitive social and behavioral problems, as well as many physical symptoms [24,56,71].

According to a study by Anagnostopoulos et al., no overall differences in frequency or type of psychiatric diagnosis were found between accompanied or unaccompanied refugee children and their Greek peers [72]; however, other studies found that refugee children are at elevated risk of mental health disorders [7,19,24,58]. It is interesting that no differences were found when a psychiatric assessment was made through a parent questionnaire, while in studies in which the educator was of the same nationality as the refugee, an increase in the rate of psychopathology was documented [72]. On the other hand, Hodes et al. supported the conclusion that the proportion of immigrants with a psychiatric diagnosis was higher than in both accompanied/unaccompanied refugees and groups of Greek children [73]. Specifically, 91% of the immigrant group received a psychosocial diagnosis, as opposed to 49% of the Greek group [72,73]. Economic migrants are reported to have low socioeconomic status, poor job status, bad living conditions, and only a low rate of health insurance coverage; these factors predispose children and adults to mental disorder [73]. However, as previously reported by Cortes, refugees lack the option of emigrating back to their homeland after prolonged periods of living in the host country, and hence, may be more inclined to invest in human capital [74]. This may take the form of becoming naturalized citizens, improving language skills, and being more likely to assimilate to the earnings growth path of the native-born population [74]. On this basis, the proportion of immigrants with a psychiatric diagnosis may be higher than currently accepted estimates among refugees [74]. However, different sources may provide conflicting data on the higher rate of psychopathology in immigrants [72,73]. Conversely, refugees report problems regarding their general health status, such as their difficulty in obtaining access to public healthcare services [72]. 

It is remarkable that many parents do not send their children to school, since they think that this could lead to permanent residence in Greece [72,75]. In 2017, the Ministry of Education, Research, and Religious Affairs, and the Greek mission of the International Organization for Migration (IOM) implemented a number of educational policies by supplying children with necessary school equipment [73]. According to the Ministry of Education, Research, and Religious Affairs, it was estimated that only 2500 of 12,000 school-age children enrolled in schools throughout Greece since the start of 2017, both in primary and secondary education [72,73,76].

#### 2.2.2. Physical Health and Immunization Status of the Child and Adolescent Refugees

Furthermore, nearly one-third of the child population presented clinical laboratory abnormalities requiring intervention, which also varied by age and origin. Eosinophilia, anemia, and low ferritin are often observed in screening tests [77,78]. Dental problems are the most frequently reported health issue. In addition, skin, respiratory, and surgical diseases were reported in refugee children [77]. Moreover, various forms of malnutrition were recorded in children, particularly in infants, as breastfeeding is a challenge for mothers during their journey [79].

In addition to routine carrier testing, carrier screening for multi-drug resistant tuberculosis (MDR-TB) should be performed in refugees and migrants upon admission to a healthcare facility [80]. Greece is one of the EU countries that perform screening for active and latent TB, and TB prevalence is believed to be low [81].

It is most important that an increase in the mortality of unaccompanied children was reported in the peak migrant period of 2014–2015 compared to the previous years of 2009–2013, when no deaths were reported [58]. This rise results from children who were admitted to hospital for medical care and eventually died, or who were admitted dead in order to determine the cause of death [58]. This finding raises serious ethical and social questions.

The majority of refugee children lack proof of immunization [77]. For instance, 80% of individuals have unknown vaccination status and suboptimal serological protection against HBV. However, no child was reported to be suffering from chronic HBV or hepatitis C virus (HCV) infection. On the other hand, four-fifths of laboratory-confirmed symptomatic cases of hepatitis A virus (HAV) infection recorded in an eight-month period in 2016 were in Syrian children under 15 years in hosting facilities [82].

## 3. The Refugee Crisis Challenges the Greek Healthcare System

### 3.1. Barriers to Health System Access

Pursuant to Article 33 of the National Law 4368/2016, uninsured and vulnerable social groups, such as asylum seekers and members of their families, are entitled free of charge to necessary health, pharmaceutical, and hospital care. However, refugees face administrative barriers in access to healthcare, which are linked to the failure of the authorities to provide a Social Security Number (AMKA) [83,84,85,86]. Following a joint statement by 25 NGOs in August 2017 [85], a circular was issued on 13 February 2018 to clarify the process of issuing AMKA to beneficiaries of international protection and asylum seekers [85]. The Greek government currently provides NHS services free of charge for refugees [17,87,88,89].

Moreover, the economic crisis had a direct effect on the health sector [10]. Primary and secondary care are suffering from major shortages in crucial equipment and technically equipped staff, unmet health needs, and ineffective central coordination [10]. The ongoing refugee crisis poses additional threats to the NHS. Both natives and foreigners are facing barriers in using healthcare services [10]. 

#### 3.1.1. Primary Healthcare Services for Refugees and Other Migrants

The Greek NHS was profoundly affected by the synergy of the economic and refugee crises, as reflected in the Aegean islands [35,90,91,92]. Without doubt, the reception centers in the Greek islands, already overstretched by the impact of the long-lasting economic crisis, witnessed the arrival of a large number of refugees; thus, they were then ill-prepared to cope with this influx and to address both locals’ and refugees’ needs [91,92]. According to a cohort study by Hermans et al. with 2291 patients, there is an urgent need for mental [35,90] and dental healthcare at Lesvos Island, which received almost half of the migrants who entered Europe [35]. More specifically, it was recently reported that most refugees (up to 80%) who were referred to a psychologist are enrolled in ongoing psychological care, and 30% of these need psychiatric support [91]. However, many patients on the islands wait three to six months for appointments with the psychiatrist, while, in some cases, patients with severe mental illness are detained at the police station’s jail, where there are no equipped staff for emergency responses [92]. Blitz et al. reported that refugees have less access to necessary healthcare as only just over one-quarter of participants surveyed (26%) stated that they had access to psychological services [8,93,94]. 

Furthermore, there is evidence that medical assessment may be especially difficult to achieve in a refugee camp [95]. An investigation of a shigellosis outbreak in a refugee camp in Greece showed that the outbreak size was underestimated due to difficulties in language, under-diagnosis of cases with mild symptoms, or denial of symptoms from patients unwilling to risk a delay in departure [96]. In addition, it was reported that a full set of vital signs were measured in fewer than 5% of patients; thus, the severity of the disease remained largely unknown [28,76]. Moreover, the health services for infectious diseases that are provided to refugees and asylum seekers are highly fragmented [95]. Data collection is often missing or inefficient between or within countries, and this can directly impair the response to public health emergencies [79]. Records were only kept in 11% of consultations that met the case criteria for clinical surveillance reporting, as based on an independent assessment [79]. The Greek Ministry of Health is currently establishing a syndromic epidemiological surveillance system to detect potential health emergencies and infectious threats among refugees in accordance with experience from other European countries, such as Italy [97].

The lack of adequate cultural mediators is also a major factor hampering access to care. Interpreting was provided by volunteers; however, professional interpreters were demanded in order to enhance confidentiality [17,87]. Greek social workers are now added to primary healthcare (PHC) settings. Their role is to create an entry to secondary healthcare and Greek emergency medical services (EMS) [17,87]. Difficulties in the referral system were overcome when Greek-speaking social workers were employed. Their responsibility is to contact the social service of the hospitals and to promote the well-being of the refugees [88,89,95].

There are also transportation problems [88,89,95]. According to Greek law, refugees are not allowed to be transported by private transport until they are issued with international protection applicant cards; thus, no one takes the responsibility of driving them to hospitals [88,89,95]. A non-emergency medical carrier for transportation, such as a bus-based public transport system, is one possibility, although this is not available for every camp. After receiving an international protection applicant card, refugees are driven to the local hospitals by private cars and buses [88,89,95]. However, for emergencies, hospital ambulances are called by the doctor or the manager of a hotspot (often a military or police officer). There are evidently too many calls, and this may delay the delivery of service [88,89,95].

#### 3.1.2. Secondary Healthcare Services for Refugees and Other Migrants

In addition, the sudden influx of refugees exposed critical public health issues, including ineffective emergency responses to address humanitarian needs, as well as the provision of health and social protection [88,89,95]. It was reported that Greek hospitals are struggling to meet the demands of both local people and migrants, mainly due to the lack of medical and human resources amid the economic crisis [10,16,17,18]. The health of refugees and migrants is jeopardized by barriers in access to Greek EMS [10,16,17,18]. Most of the barriers are related to language, culture, and lack of information about the healthcare system in the host country [10,16,17,18].

Linguistic and cultural differences make it more difficult to assess and manage these problems [10,16,17,18]. In consequence, migrants are often under time pressure [10,16,17,18]. Their care is often uncoordinated and they face difficulties in accessing proper specialized healthcare [10,16,17,18]. Frequent interruptions and multiple simultaneous consultations are likely to impair the quality of the consultations, but do reflect the reality of the working environment [18]. In all healthcare facilities, but especially in public hospitals, translation services and feedback mechanisms to enhance communication are urgently needed [10,16,17,18]. In addition, lack of continuity of care is a crucial issue (no personal hospitalization or medical history, or only in the local language), as well as difficulties in obtaining proper medication during the journey [10,16,17,18]. 

It is important that there was recently a shift of attention from the vulnerable victims of the Greek economic turmoil to the refugees, and this annoyed some members of the local population. In parallel with support for the refugees, it is essential to provide assistance to Greek citizens, thus developing a future balance between society’s integration, humanity, and security [34].

### 3.2. Trends in Healthcare Resource Utilization

Although some refugees refused to receive any care because they wanted to continue their journey as soon as possible, the majority reported that they received inadequate information about the rules in the holding centers, as well as on the organization and location of health services [38].

Most of the barriers faced are related to cost, language, and lack of information about the healthcare system in the host country [32,38]. Cultural barriers to accessing healthcare were more rarely mentioned. This was predominantly by female participants, who preferred doctors of the same gender and geographical/cultural background [32,38]. However, in cases of emergencies, the gender of the doctor was considered less important [32,38].

It is apparent that differences in language are problematic in all settings for both healthcare professionals and refugees. It is too difficult to overcome this problem, even when interpreters are available.

Refugees also argued that they had difficulties in accessing medical care at busy border crossings and long-term reception centers [32,38], and the local customs and administrative problems of the healthcare system hampered accessibility. Furthermore, financial difficulties in making out-of-pocket payments for health and social care services are reported as an issue among refugees [34]. Additional barriers were linked to lack of time and continuity of care; this was related to the specific setting in hotspots, transit centers, and hospitals [32,38]. The bias of these groups toward the operation of public services may frighten some migrants [34,97,98].

### 3.3. Expenditure Data across the Health System

Since 2009, the Greek economy was severely harmed by a national debt crisis. The main causes of this debt crisis were related to continuous government deficits and inaccurate statistics, as well as to structural problems in the country’s public and private sector [98,99,100]. The symptoms of the crisis were expressed through the risk of borrowing funds at very high interest rates, the possibility of debt default, and threats to the stability of the Eurozone. These symptoms were faced through a series of lending agreements (Memorandums of Understanding) with the so-called troika: the International Monetary Fund (IMF), the European Central Bank (ECB), and the European Commission (EC) [101]. These agreements, however, included a series of harsh austerity measures, sudden reforms, deep budget cuts, large tax increases, and numerous privatizations that led to impoverishment for a large part of the population and loss of income and property [102,103].

Moreover, the country’s gross domestic product (GDP) fell from 236 billion in 2008 to 184 billion in 2016, a value loss of 22%, while the unemployment rate of the workforce rose from 7.3% on 2008 to 20.1% in 2017 [104]. According to estimates made by Eurostat in 2015, 35.7% of the population was at risk of poverty or social exclusion [105]. The crisis also negatively affected health expenditure in Greece and the health status of the population. Total health expenditure in the country fell from €22.49 billion in 2009 to €14.73 billion in 2015, while the mean per capita healthcare expenditure declined from €2024 in 2009 to €1361 in 2015, an overall decrease of almost 33% [106]. The economic recession also negatively affected the health status of the population in Greece [100,107,108]. Finally, it must be mentioned that, whilst Greeks theoretically have access to healthcare and treatment in public hospitals, in reality, access is difficult and lengthy due to a general lack of infrastructure, equipment, and medical and human resources [16,17]. This is why high out-of-pocket private spending on health is a marked feature of the Greek healthcare system and continues to rise. In 2015, out-of-pocket payments comprised over one-third (35%) of total health spending, more than double the EU average (15%) [109].

A further burden put on to the already heavily loaded Greek health system comes from the thousands of refugees who pass the Greek borders. Even though most of the cost of refugees’ healthcare is incurred by NGOs in order to permit primary healthcare in clinics in urban areas or in camps and reception centers, it is known that hundreds of refugees are transported to public hospitals [110]. Most refugees are now living in urban areas for extensive periods of time. Hence, this model can often lead to duplication of efforts through over-referral of patients by NGOs to public hospitals, and thus, to an increased burden on the health sector. Nevertheless, there is a lack of recorded data on the number of refugees who visit Greek hospitals to seek medical treatment [10].

According to the Ministry of Immigration Policy and a report from the Bank of Greece, the estimated cost of the refugee crisis to public expenditure for 2016 was about 0.3% of the country’s GDP (i.e., about 600 million euros), and 35.7% of this sum was spent on open reception facilities, 26.3% on research and rescue operations, 20.6% on first reception facilities, 8.1% on transfers, 6.5% on asylum and relocation, and 2.8% on returns [111]. No exact cost for refugee health expenditure was made available by the Greek government. A report from the government’s General Secretariat referred to 42,787 vaccinations of refugee children that took place from May 2016 to January 2017 with the collaboration of the Ministry of Health and various NGOs. The cost of these vaccinations, however, was at least partly covered by the NGOs [112].

There is still only limited information on the cost of refugees’ healthcare in other countries. In comparison to the regularly insured, a study in Germany found that asylum seekers had more hospital and emergency department admissions that could be avoided through good outpatient care or prevention, and that their average expenditures were 10% higher. The authors concluded that access to the healthcare system, especially outpatient and mental healthcare, could improve asylum seekers’ health status and integration, possibly at lower costs [113]. The lesson from past migrations is that restricting healthcare is economically counterproductive [114].

In conclusion, the economic effects of the recent refugee crisis on healthcare spending in Greece are not clear. Perhaps, however, someone should take into account the economic impact of refugees in the countries where they arrive. Importantly, even though in the short term they put extra weight on a country’s budget, in the medium-to-long term, successful and timely integration of refugees into the labor market can contribute to greater flexibility, help address demographic challenges, and improve fiscal sustainability [109]. In this process, integration is the key. Nevertheless, what is clear from previous research and literature is that the earlier and better the integration, the more likely it is that legally residing, third-country nationals will make a positive contribution to growth and public finances [115]. However, the short-, medium-, and long-term impacts are bound to differ across countries, not only because of differences in the size of inflows, but also on whether or not a migrant passes through or stays, and on whether or not he is granted protection status or is rejected, together with the individual’s profile, as well as the host country’s economic structure and capacity to integrate those that will be granted protection [115].

### 3.4. The Role of Non-Governmental Organizations

As the broader Greek public healthcare sector cannot adequately handle the massive influx of refugees, a volunteer movement arose in order to help. This includes various NGOs, other social groups, and many individuals [116]. International organizations include the United High Commission for Refugees (UNHCR), the International Committee of the Red Cross (ICRC), the International Organization of Migration (IOM), and large international NGOs such as MSF, Médecins du Monde (MDM), and Save the Children, as well as many local ad hoc grassroots organizations, which quickly deployed staff and services to meet the needs of the refugees [117].

In fact, in most camps, primary healthcare (PHC) is generally ensured by army doctors and international and Greek NGOs, and these play a critical role in delivering healthcare services in all sites [17]. Since recently, medical services were provided mainly by a general practitioner and a nurse. Nursing, which is offered by NGOs and international establishments such as the Red Cross, provides basic services ranging from primary care to health promotion in the shelter camp [116]. Gynecologists (preferably female), midwives, dentists, psychologists, and psychiatrists were lately included in the camp clinics [17].

It is, therefore, important to consider the current roles of NGOs, which may have to be extended in order to provide appropriate healthcare services to migrants, refugees, and asylum-seekers, particularly when these groups are excluded from the public health system [88].

Access to specialist care or treatment in about half of all cases is offered by NGOs and not by the NHS [55]. NGOs also have a distinct role to play in emergency preparedness. Serving, negotiating, debating, monitoring, reporting, lobbying, or supporting NGOs are constantly seeking to achieve adequate and effective standards of law, policy, and practice [35]. In these terms, NGOs are key players in advocating adequate protection in the context of healthcare provision. Likewise, medical screening, and more research and systematic data collection by NGOs are needed to inform policies and to support the development of strategies for the specific humanitarian challenges posed by the recent refugee waves reaching Greece [55]. On the other hand, the existence of NGOs perpetuates the government’s reliance on them, and this is a matter of great concern. The NHS should be able to integrate asylum seekers and refugees.

## 4. Impact of the Refugee Crisis on the Well-Being of Rescue Workers

‘‘Rescue workers’’ or ‘‘rescuers’’ are defined as the professionals or volunteers who engage in stressful activities targeted at providing assistance to people in emergency circumstances [118]. It is totally clear that the refugee crisis had a negative impact on rescue workers’ mental health. As with disaster victims and refugees, rescue and caregiving personnel may be at high risk of psychological impairment and PTSD, especially without adequate training or psychological support [118]. Many significant factors were identified as predictors for PTSD, perceived burnout, and well-being, including the family status (higher in single, divorced personnel) and increasing age, as well as the duration of demanding situations, such as gathering dead bodies. Female rescue workers were at significantly greater risk of PTSD [118].

PTSD is further accompanied by exhausting working conditions and lack of continuous psychological support. In other words, PTSD is positively correlated with burnout and inversely correlated with well-being.

Pre-departure psycho-educational training, as well as periodically organized psychological support sessions, might be essential for the prevention and mitigation of psychiatric morbidity in caregivers in refugee hotspots [119,120,121].

## 5. Conclusions

Greece is spoken of not only as a space of settlement and transit for refugees and immigrants, but also as a space for work and retirement. However, the refugee crisis had a major impact on the Greek NHS.

The recent steep influx of forcibly displaced people settling in Greece raised critical issues concerned with health policy. The health system must be ready to provide an effective response to the many challenges, especially in the prevention and control of the transmission of communicable diseases, and the treatment of acute infectious diseases by achieving effective health screening and vaccination coverage [95,122]. The results of medical screening can help identify health risk factors and epidemiological characteristics, and they should subsequently lead to more effective prevention and provision of healthcare services, and to guiding policy and interventions by authorities dealing with the specific needs. Therefore, it is important to establish a simple but accurate disease surveillance system [98].

Furthermore, the government should develop a strong NHS which can cater for the needs of populations facing distinct challenges, by empowering the NHS. The NHS in Greece must deal with traumatic experiences, and cultural and linguistic differences, as well as gaining trust, which requires more than basic care. Protection of fundamental human rights and refugee laws is urgently needed; this must offer a comprehensive approach combining a humanitarian and political response [123,124]. Furthermore, priorities include the provision of care for mental comorbidities via psychosocial training for healthcare providers, while people should be helped to retain their sympathetic approach to refugees.

Child-focused research could shed light on the multidimensional factors that contribute to children’s mental illness [123,124]. Social intervention strategies to fight sexual exploitation of adolescents and children constitute a major social intervention. The prevalence of psychopathology is high, and it is certainly preferable to treat these not only on clinical and humanitarian grounds, but also on cost-effectiveness grounds [123,124].

Effective approaches are recommended to encourage young unaccompanied minors to speak about their difficulties and to support them [22]. In these terms, a collective narrative methodology called the “Tree of Life”, originally developed by Ncube-Mlilo and Denborough, constitutes a culture-dependent eight-hour workshop as a paradigm to promote children’s mental health [22].

The National and Kapodistrian University of Athens is developing the actions needed and the approach to meet the healthcare problems. This involves the participation of more than 100 doctors and scientific bodies in order to offer immediate and coordinated volunteer actions, including administrative support, healthcare services, and supplies [75]. More recently, Lionis et al. reported methods used for enhancing PHC for refugees in the context of a structured European project [49]. This work plan includes the assessment of the health needs of all the people reaching Europe, and it is anticipated to promote the working conditions and satisfaction of healthcare workers, as well as the interaction and collaboration between refugees, healthcare workers, and host communities [49].

As reception and holding centers are overcrowded, it is essential to have guidelines and instruments for accurate initial assessment (screening triage) and to provide transport to definitive care for refugees and other migrants [10,34,98]. For specialized human-centered, gender-specific care, it is essential to incorporate female healthcare providers and interpreters into medical teams [10,34,98]. To strengthen the referral mechanisms, NGOs and Greek health authorities should communicate strategically, in order to facilitate the transition of health service delivery to the Greek healthcare system [10,34,98]. Moreover, well-organized medical care adapted to the complex needs of populations should be flexible and cost-effective, and should protect local health structures from being overwhelmed at entry locations. Such an approach is essential for protecting social coherence by minimizing the impact on local public infrastructures [10,34,98].

On the other hand, Greek healthcare faces many severe challenges. The ability to deliver efficient management, adequate finances, and human resources is a pre-requisite. Greece is still bearing the brunt of the economic crisis. The economic downturn led to significant shortages in crucial equipment and unmet health needs due to economic limitations, which affected both locals and refugees. For this reason, wealthy nations should also push for measures to help the economically distressed countries of southern Europe to cope with the refugees that people in the north are not willing to accept.
